# Red Carrot Cells Cultured *in vitro* Are Effective, Stable, and Safe Ingredients for Skin Care, Nutraceutical, and Food Applications

**DOI:** 10.3389/fbioe.2020.575079

**Published:** 2020-10-21

**Authors:** Martino Bianconi, Laura Ceriotti, Salvatore Cuzzocrea, Emanuela Esposito, Giovanna Pressi, Elena Sgaravatti, Oriana Bertaiola, Chiara Guarnerio, Elisa Barbieri, Alessandra Semenzato, Stefano Negri, Mauro Commisso, Linda Avesani, Flavia Guzzo

**Affiliations:** ^1^Demethra Biotech Srl, Vicenza, Italy; ^2^VitroScreen Srl, Milan, Italy; ^3^Department of Chemical, Biological, Pharmaceutical and Environmental Sciences, University of Messina, Messina, Italy; ^4^Department of Pharmaceutical and Pharmacological Sciences, University of Padua, Padua, Italy; ^5^Department of Biotechnology, University of Verona, Verona, Italy

**Keywords:** carrot cell cultures, anthocyanins, antioxidants, inflammation, reconstructed human skin models, skin aging, cosmetics, nutraceuticals

## Abstract

Plant biomasses growing in bioreactor could be developed as production systems for cosmetic ingredients, nutraceuticals and food additives. We previously reported that the red carrot cell line R4G accumulates high levels of anthocyanins, which are potent antioxidants with multiple health-promoting properties. To investigate the industrial potential of this cell line in detail, we tested extract for antioxidant and anti-inflammatory activity in the mouse monocyte/macrophage cell-line J774A.1 and in reconstructed skin tissue models. We also compared the R4G extract to commercial carrot extracts in terms of stability and metabolomic profiles. We found that the R4G extract have potent antioxidant and anti-inflammatory activities, protecting mammalian cells from the oxidative stress triggered by exposure to bacterial lipopolysaccharides and H_2_O_2_. The extract also inhibited the nuclear translocation of NF-κB in an epidermal skin model, and induced the expression of VEGF-A to promote the microcirculation in a dermal microtissue model. The anthocyanins extracted from R4G cells were significantly more stable than those found in natural red carrot extracts. Finally, we showed that R4G extract has similar metabolomic profile of natural extracts by using a combination of targeted and untargeted metabolomics analysis, demonstrating the safety of R4G carrot cells for applications in the nutraceutical and food/feed industries.

## Introduction

Plant cell and tissue culture, which history can be traced back to the 30s of the 20th century (Thorpe, 2007), offers an attractive platform for the production of cosmetic, nutraceutical and food ingredients, with many advantages over agricultural production methods (Eibl et al., 2018). First, these plant biomasses growing in bioreactor are season-independent, allowing year-round manufacturing. Second, they are more environmentally sustainable than traditional agriculture, which requires land, consumes water resources, generates greenhouse gases, and may require the application of herbicides and pesticides. Third, plant cells are grown aseptically in fully-defined culture media, so the resulting products are sterile and free of environmental pollutants. Finally, plant cells grown *in vitro* retain the ability of the source plant to accumulate not only primary metabolites but also certain classes of specialized secondary metabolites with health-promoting activities. For example, plant cells are rich sources of polyphenols, which have antioxidant and anti-inflammatory activities that make them suitable as cosmetic and nutraceutical ingredients to inhibit skin aging and reduce the risk of diseases linked to oxidative stress (Cory et al., 2018).

Skin aging is a complex biological phenomenon reflecting intrinsic and extrinsic factors that trigger the progressive loss of structural integrity and physiological functions (Shin et al., 2019). Intrinsic aging involves inflammation-related damage caused by the accumulation of reactive oxygen species (ROS) over time, whereas extrinsic aging is caused by multiple factors that increase ROS levels, including exposure to UV light, pollution, smoking, a poor diet, poor sleep, and other lifestyle components. Ultimately, these combined effects reduce skin elasticity, promoting the development of wrinkles and progressive dermal atrophy (Farage et al., 2008). In the skin, 1.5–5% of consumed oxygen is converted into ROS as byproducts of aerobic metabolism (Kammeyer and Luiten, 2015) leading to the peroxidation of intercellular lipids and cell membrane phospholipids (Quan et al., 2009). The liquid-crystalline lipid structures of the cell are disrupted, inhibiting epidermal barrier activity and increasing *trans-*epidermal water loss. Dehydration of the stratum corneum may weaken the epidermal barrier, leading to further dehydration and greater susceptibility to irritants, allergens, and the overall aging processes (Bravo et al., 2016).

The molecular mechanism of ROS-mediated inflammation during aging is partially based on the activation of transcription factors and signaling proteins that regulate the proteolytic degradation of the extracellular matrix (ECM) in the skin (Pillai et al., 2005). For example, aging causes an increase in the expression of matrix metalloproteinases (MMPs), the ubiquitous endopeptidases that degrade ECM proteins (Shin et al., 2019). This may be induced by the activation of nuclear factor κB (NF-κB) and the secretion of tumor necrosis factor α (TNFα) and other pro-inflammatory cytokines such as interleukin (IL)-1, IL-6, and IL-8 (Briganti and Picardo, 2003; Choi et al., 2016). The skin has an antioxidant defense system comprising an interlinked network of enzymes that convert ROS to harmless water and molecular oxygen, as well as a cell-based system to resolve inflammation by promoting the secretion of anti-inflammatory cytokines such as IL-10. However, these systems begin to lose potency with age, contributing to the overall decline in skin integrity (Rhie et al., 2001). Today’s skincare products include antioxidants as anti-aging ingredients, which are proposed to scavenge ROS and/or inhibit ROS-mediated inflammation (Pillai et al., 2005).

Plants have long been a source of natural antioxidants and inhibitors of inflammation, melanin hyperpigmentation, and other skin conditions. There is consistent evidence that polyphenols can prevent oxidative damage and reduce inflammation (Hussain et al., 2016) by donating electrons and hydrogen atoms that neutralize ROS and other free radicals, or directly inhibit the lipoperoxidation chain reaction (George et al., 2009). Plants and plant cells are therefore of great interest to the cosmetics industry as a source of novel polyphenol-based anti-aging ingredients. The antioxidant and anti-inflammatory properties of polyphenols may also help to reduce the risk or severity of diseases triggered by oxidative stress. Individual polyphenol compounds and mixtures have been shown to modulate inflammatory signaling pathways *in vitro* and *in vivo*, and to inhibit carcinogenesis and tumor growth (Piccolella and Pacifico, 2015; De Silva and Alcorn, 2019). Finally, certain polyphenols not only provide health benefits but also act as natural pigments and can therefore be used as food colorings. Anthocyanins are polyphenolic compounds that confer red, purple or blue color depending on the pH, and they could therefore be used to replace artificial food colorants with a natural alternative (Zhang and Furusaki, 1999).

We previously described the selection of a pigmented carrot (*Daucus carota* L.) cell line that accumulates large quantities of polyphenols (Ceoldo et al., 2009), including eight specific cyanidin derivatives, some acylated with coumaric, caffeic, ferulic, and sinapic acids (Gläßgen and Seitz, 1992; Ceoldo et al., 2009). However, even if plant cells produce polyphenols with known antioxidant and anti-inflammatory activities, the combined effect is difficult to predict because the overall diversity and individual quantities may vary, and there may be synergistic effects among different compounds. Here we assessed the activities of an extract of the anthocyanin-accumulating carrot cell line R4G, revealing antioxidant and anti-inflammatory activities when applied to mouse J774A.1 monocyte/macrophage cells, the ability to promote the nuclear translocation of NF-κB, and the ability to upregulate vascular endothelial growth factor (VEGF-A) expression when applied to *in vitro* reconstructed human skin models. Because these activities make the carrot cell extract suitable for the development of cosmetic ingredients, nutraceutical supplements and food colorings, we compared it to natural red carrot extracts already available on the market.

## Materials and Methods

### R4G Cell Line Maintenance and Growth

The R4G cell line was maintained on solid Gamborg’s B5 medium (Gamborg et al., 1968) supplemented with 25 g/L sucrose and 3 mg/L naphthalenacetic acid. The pH was adjusted to 5.5 before autoclaving. Callus was incubated at 25°C in the dark and subcultured at 3-week intervals. Erlenmeyer flasks (1 L capacity) containing 250 mL liquid Gamborg’s B5 medium were inoculated with callus tissue representing ∼10% (w/v) of the medium. The suspension cultures were maintained at 25°C in the dark with constant orbital agitation at 120 rpm and were subcultured every 14 days. The biomass was increased by transferring inoculums to 3-L flasks containing 1 L of fresh liquid culture medium. To increase the anthocyanin content after 12 days of fermentation in flasks, the culture was introduced to a 50-L bioreactor containing 30 L of productive Gamborg’s B5 liquid medium with a higher sucrose concentration (40 g/L) and subcultured every 14 days.

### Preparation of Dry R4G Extract for Activity Testing

After 14 days of fermentation, cell suspensions were passed through a 50-μm mesh filter and the medium was discarded. The retained cells were washed with 0.9% (w/v) NaCl supplemented with 0.5–2% citric acid and then homogenized with an Ultra-Turrax device (IKA, Staufen, Germany) at 15,000 rpm for 20 min. The biomass of homogenized cells was dried using a Mini Spray Dryer (BUCHI-B290).

### Cultivation of J774A.1 Cells and Evaluation of Cell Viability

The monocyte/macrophage cell-line J774A.1 was grown in Dulbecco’s modified Eagle’s medium (DMEM) supplemented with 2 mM glutamine, 25 mM HEPES, 100 U/mL penicillin, 100 μg/mL streptomycin, 10% fetal bovine serum (FBS) and 1.2% sodium pyruvate at 37°C in a 5% CO_2_ atmosphere. The cells were seeded at a density 4 × 10^4^ per well in 96-well plates (150 μL medium per well) and were allowed to adhere for 4 h. We then refreshed the medium and exposed the cells to different concentrations of R4G extract (0.01, 0.05, 0.1, 0.5, 1, and 5 mg/mL dissolved in complete medium). The viability of J774A.1 cells in the presence of the extract was determined using a live-cell assay based on the dye MTT (3-(4,5-dimethylthiazol-2-yl)-2,5-diphenyltetrazolium bromide) as previously described (Abe and Matsuki, 2000). J774A.1 cells were incubated with 0.2 mg/mL MTT at 37°C for 1 h. The medium was removed and the cells were lysed with 100 μL dimethylsulfoxide (DMSO). The reduction of MTT to formazan was measured at an optical density of 550 nm (OD_550 nm_) using a using a Tecan (Männedorf, Switzerland) microplate reader. The same assay was used to determine the viability of J774A.1 cells stimulated with 1.0 μg/mL *Escherichia coli* lipopolysaccharides (LPSs) for 24 h or with 200 μM H_2_O_2_ for 10 min, both alone or in the presence of the extract.

### Assays for Anti-inflammatory Activity

We seeded 8 × 10^5^ J774A.1 cells into 24-well tissue culture plates and incubated them as described above until the cells reached confluence (usually 48 h). The cells were then washed twice in phosphate-buffered saline (PBS) before incubation in Ham’s F-12 medium (Ham, 1965) with 1% fetal calf serum (FCS). To test for anti-inflammatory activity, the cells were exposed to R4G extract and stimulated with 1.0 μg/mL *E. coli* LPS for 24 h before detaching them with 0.25% trypsin in 1 mM ethylenediaminetetraacetic acid (EDTA) and re-seeding at a density of 2 × 10^6^. After incubation for a further 24 h, the cells were washed three times in cold PBS, disassociated with 0.125% trypsin and centrifuged at 8000 × *g* for 5 min at 4°C. The cell pellets were resuspended in 100 μL lysis buffer (25 mM Tris-HCl pH 7.4, 1.0 mM ethylene glycol tetraacetic acid (EGTA), 1.0 mM EDTA, 0.5 mM phenylmethylsulfonyl fluoride (PMSF), 100 mM Na_3_VO_4_, 10 μg/mL aprotinin, 10 μg/mL leupeptin, 10 μg/mL pepstatin) and incubated on ice for 20 min. The lysate was centrifuged for 15 min as above and the protein concentration in the supernatant was determined using the Bio-Rad protein assay (Bio-Rad, Hercules, CA, United States).

The proteins were fractionated by sodium dodecylsulfate polyacrylamide gel electrophoresis (SDS-PAGE) and transferred onto a polyvinylidene difluoride (PVDF) membrane at 0.26 mA for 30 min. The expression levels of IκBα, NFκB, iNOS, and COX-2 were determined by probing the membrane with the corresponding primary antibodies in PBS 1X containing 5% not fat dry milk and 0.1% Tween-20, pH 7.4 (PMT), overnight at 4°C: anti-IκBα (sc1643; Santa Cruz Biotechnology, Dallas, TX, United States) diluted 1:500, anti-NFκB (Purified Mouse Anti- NF-κB Colne20/p65; BD Biosciences, San Jose, CA, United States) diluted 1:1000, anti-iNOS (sc8310; Santa Cruz Biotechnology) diluted 1:500, and anti-COX-2 (sc376861; Santa Cruz Biotechnology) diluted 1:500. The next day, the membranes were rinsed with PBT and incubated with a specific alkaline peroxidase (AP)-conjugated secondary antibody diluted 1:5000 in PMT (Thermo Fisher Scientific, Waltham, MA, United States) for 1 h at room temperature. The results were expressed as integrated intensities relative to controls; we used β-actin as a control for protein loading, probed with the corresponding antibody diluted 1:1000 (Santa Cruz Biotechnology). Signals were detected by enhanced chemiluminescence (ECL) detection according to the manufacturer’s instructions (Thermo Fisher Scientific). The relative expression of the protein bands was quantified by densitometry with Bio-Rad ChemiDoc XRS Plus software. Images of blot signals (8 bit/600 dpi resolution) were imported to Image Quant TL (v2003) for analysis.

### Enzyme-Linked Immunosorbent Assay (ELISA)

Samples of medium from the J774A.1 cells stimulated with LPS were used for the detection of TNFα and IL-1β by TNFα and IL-1β Mouse ELISA Kits (Invitrogen, Carlsbad, CA, United States) according to the manufacturer’s recommendations. Briefly, clear polystyrene microplates were covered with monoclonal anti-mouse TNF-α or IL-1β antibodies (100 μl/well, 20 μg/ml) by incubating overnight at room temperature. Then the wells were aspirated and washed with Wash buffer (0.05% Tween 20 in PBS, pH 7.4) 5 times (the aspiration and washing with the same buffer was repeated before each following addition), and the plates were blocked with 300 μl per well of blocking solution (PBS containing 1% BSA and 5% sucrose) for 2 h at room temperature. Then, 50 μl of the blocking solution was added to each well followed by the addition of 100 μl per well of samples or standards diluted in PBS (the standards from ELISA Kit were used) and the plates were incubated 2 h at room temperature. After this, 100 μl of biotin anti-TNF and anti- IL-1β (2 μg/ml of the blocking solution) were added to each well and incubated for 2 h at room temperature, followed by a 20 min incubation with 100 μl (2 drops) per well of streptavidin-HRP ready-to use solution. Finally, 100 μl/well substrate solution (0.05% 3,3′,5,5′-tetramethylbenzidine and 0.012% H_2_O_2_ in 0.05M citrate buffer, pH 5.0) was added and incubated for 30 min at 37°C. Absorbance was read at 450 nm using a Tecan microplate reader and background wavelength correction was set to 540 or 570 nm.

### Measurement of Nitrite Levels

Total nitrite levels, as an indicator of nitric oxide synthesis, were measured in the supernatant of J774A.1 cells stimulated with H_2_O_2_. Briefly, nitrate was reduced to nitrite by incubation with 670 mU/mL nitrate reductase and 160 mM β-nicotinamide adenine dinucleotide 3-phosphate at room temperature for 3 h. The total nitrite concentration in 100-μL samples of medium was then determined using the Griess reaction by adding 100 μL of Griess reagent, a 1:1 ratio of 0.1% (w/v) *N*-(1-naphthyl) ethylenediamine dihydrochloride in H_2_O and 1% (w/v) sulfanilamide in 5% (v/v) concentrated H_3_PO_4_. The OD_550_ was measured on a Tecan microplate reader. Nitrite concentrations were calculated relative to sodium nitrite standards prepared in H_2_O.

### Measurement of Malondialdehyde (MDA) Levels

J774A.1 cells were seeded at a density of 1 × 10^5^ cells/well into poly-L-lysine-coated six-well plates and cultured as described above. MDA levels were determined using the Lipid Peroxidation (MDA) Assay Kit (Colorimetric/Fluorometric) (Abcam, Cambridge, United Kingdom) as previously described (Meng et al., 2014).

### Measurement of Intracellular ROS Levels

The quantity of intracellular ROS was determined using the Total ROS/Superoxide detection kit (Enzo Life Sciences, Farmingdale, NY, United States) as previously described (Meng et al., 2014). After pre-treatment with extract, J774A.1 cells were trypsinized as described above before washing twice with the washing buffer provided in the kit. The cells were then incubated with 10 μM 5-(and-6)-carboxy-2′,7′ dichlorodihydrofluorescein diacetate (carboxy-H2DCFDA) at 37°C in the dark for 30 min before fluorescence detection on a on a Tecan microplate reader (absorption peak at 504 nm, emission peak at 524 nm). The level of intracellular ROS was expressed as a percentage relative to the control (untreated cells, nmol/mL).

### Statistical Analysis

All assays were carried out at least three times and the data are presented as means ± standard errors of the mean. Statistical significance was established by one-way analysis of variance (ANOVA) followed by Bonferroni *post hoc* correction for multiple comparisons, with a threshold of *p* < 0.05.

### Generation, Cultivation, and Maintenance of Reconstructed Human Epidermis (RHE)

RHE/S/17 (Episkin, Lyon, France) in a 0.5-cm^2^ format was produced from normal human keratinocytes, reconstituted by airlift culture on polycarbonate filter inserts in chemically defined medium for 17 days. The RHE batch was tested to confirm the absence of HIV1, HIV2, Hepatitis B virus, Hepatitis C virus, and mycoplasma. Immediately after the RHE was delivered, we confirmed the shipment integrity, color and temperature of the agar medium used for transport as per the batch-release data sheets. RHE samples were then removed from the agarose nutrient solution in a fume cabinet under sterile air flow. The inserts were rapidly transferred to six-well plates containing 1 mL/well specific maintenance medium provided by the tissue supplier at room temperature and were incubated at 37°C in a 5% CO_2_ atmosphere with saturated humidity overnight.

### RHE Treatments and Immunohistochemistry

Triplicate RHE samples from the overnight culture were treated using topical and systemic exposure protocols. Topical treatment involved the application of 15 μL saline solution containing 3% glycerol extract or 0.1% dehydrated extract to the RHE medium on the tissue surface, followed by overnight incubation at 37°C in a 5% CO_2_ atmosphere with saturated humidity. The systemic treatment involved the addition of 1 mg/mL of the extract to the RHE medium followed by overnight incubation as above. The RHE samples were then exposed to 2 MED UV light using an Oriel solar simulator with a xenon arch lamp and filter WG320. As a negative control, RHE samples were cultivated without treatment or UV irradiation. The samples were collected 4 h after irradiation, washed with 1 mL saline solution, fixed in formalin and embedded in paraffin for immunostaining with an anti-NFκB antibody (AB16502; Abcam, Cambridge, United Kingdom). The signal was visualized with the secondary antibody Alexa 555 and nuclei were counterstained with DAPI. Stained sections were visualized on a Leica (Wetzlar, Germany) DM 2500 microscope (40× magnification) and images were captured using a Leica DFC 450C camera and LASX v3.0.1 software. The signal was quantified using Fiji software. For NF-κB, the number of nuclei showing signal translocation was determined for three tissue sections per replicate and was expressed as the average number of nuclear translocations observed per field.

### Human Microdermal Endothelial Cell (HMEC) Model

The HMEC model was produced by cultivating human primary dermal fibroblasts from a donor aged 60 years (Tebu-Bio, Magenta, Italy) with human microvascular dermal endothelial cells (Lonza, Basel, Switzerland) as recommended (VitroScreen, Milan, Italy). Following tissue formation, the model was transferred to GravityTRAP plates and 70 μL of CnT-PRF medium (Prime Fibroblast medium, CELLnTEC, Bern, Switzerland) was added to the GravityPLUS plate wells (InSphero, Schlieren, Switzerland).

### HMEC Treatments and Gene Expression Analysis

The medium was removed from each well and replaced with 80 μL fresh CnT-PRF medium containing 0.1 or 0.03% dehydrated extract. The supplemented medium was filter-sterilized (0.22-μm filter) before transfer to avoid contamination. The microtissues were incubated for 72 h before lysis and RNA extraction using the RNAqueous kit (Thermo Fisher Scientific). RNA was eluted in RNase-free water, quantified by spectrophotometry and stored at −80°C. First-strand cDNA was synthesized using the High Capacity cDNA Reverse Transcription kit (Thermo Fisher Scientific) and a TaqMan assay for VEGF-A gene expression (probe VEGFA Hs00173626_m1) was carried out using an Applied Biosystems 7500 Fast Real Time PCR instrument. The change in expression in pools of 10 microtissue samples was measured against an untreated calibrator sample. Fluorescence data were collected by the internal software (SDS v2.0.6) and the raw data (RQ study results) were exported in Excel.

### Targeted and Untargeted Metabolomics

Fresh R4G cells were collected by filtration and powdered in liquid nitrogen. Three commercial products (two powdered carrot extracts and one carrot juice, named CP1, CP2, and CP3, respectively) were used as comparators. The heterogeneous samples (fresh cells, dried carrot powders, and carrot juice) were extracted and/or diluted in different ways to obtain extracts with similar final concentrations of anthocyanin for high-performance liquid chromatography diode array detector mass spectrometry (HPLC-DAD-MS) as reported in Table 1.

**TABLE 1:** Sample preparation details for targeted and untargeted metabolomics analysis.

**Materials**	Volume of extraction solvent (w/v, v/v*)	**Dilution**	**Fresh weight to dry weight conversion**	**Total dilution factor**
R4G fresh cells	3	1:10	17.5	525
commercial product 1, CP1	15	1:10	-	150
commercial product 2, CP2	50	1:10	-	500
commercial product 3, CP3	100*	1:3	-	300

Cold 99:1 (v/v) methanol/HCl was used as the extraction solvent. After careful mixing and incubation for 30 min on ice, the cell debris was removed by centrifugation and the extracts were diluted with LC-grade water and passed through a 0.2-μm pore filter for sterilization (Sartorius, Göttingen, Germany).

Targeted HPLC-DAD and untargeted LC-MS metabolomics analysis was carried out as described by Commisso et al. (2017). For targeted analysis, 30-μL samples were injected and the absorption spectra were recorded over the range 180–600 nm using 32 Karat Workstation v7.0 (Beckman Coulter, Brea, CA, United States). Anthocyanins and caffeic acid derivatives were quantified based on their UV/Vis absorbance spectra at 520 and 320 nm and were expressed as cyanidin-3-*O* glucoside and caffeic acid equivalents, respectively, by comparison with calibration curves based on authentic standards (Extrasynthese, Genay, France). For untargeted analysis, 30-μL samples were injected and the LC–MS files were processed with MZmine^1^. The resulting data matrix was submitted to Simca v13.0.3.0 (Umetrics, Sweden) for multivariate statistical analysis as previously described (Dal Santo et al., 2016). Metabolites were identified by comparing *m/z* values and MS/MS or MS^3^ fragmentation trees with an in-house library of spectra representing authentic standards or data from the literature. Identifications were confirmed by high resolution ultra-high performance liquid chromatography mass spectrometry (HR-UPLC-MS) following the injection of 3-μL samples as previously described (Commisso et al., 2019). Briefly, 200 mg of R4G fresh cell powder or 10 mg of R4G dry powder was extracted with 5 and 100 volumes, respectively, of 99% methanol in HCl (v/v) by sonicating for 10 min at 40 kHz in a Sonica Ultrasonic Cleaner (SOLTEC, Milan, Italy). The extract were centrifuged at 14,000 × *g* for 15 min and the supernatants were diluted 1:100 with LC–MS grade water (Honeywell, Charlotte, NC, United States) and passed through a Minisart RC4 filter (Sartorius) before injection.

### Stability of Anthocyanins

R4G extract were compared to anthocyanin-rich commercial extracts intended as coloring agents (CP4 – natural red carrot juice, CP2 and CP5 – powdered red carrot extracts, and CP6 – powdered natural elderberry extract). Each sample was suspended in acidified methanol (99% methanol, 1% 1M HCl) to achieve a similar absorbance at 520 nm. Aliquots of each extract were stored at 4, 22, or 32°C in the dark, and the color of the extracts was assessed over the next 98 days through the spectrophotometric absorbance at 520 nm. Three independent replicates were measured for each sample at each time point, and the mean absorbance was expressed as a percentage of the initial absorbance ± standard deviation.

## Results

### R4G Extract Show Potent Anti-inflammatory and Antioxidant Activity in Mouse J774A.1 Cells

#### Anti-inflammatory Activity of R4G Extract

**FIGURE 1:**
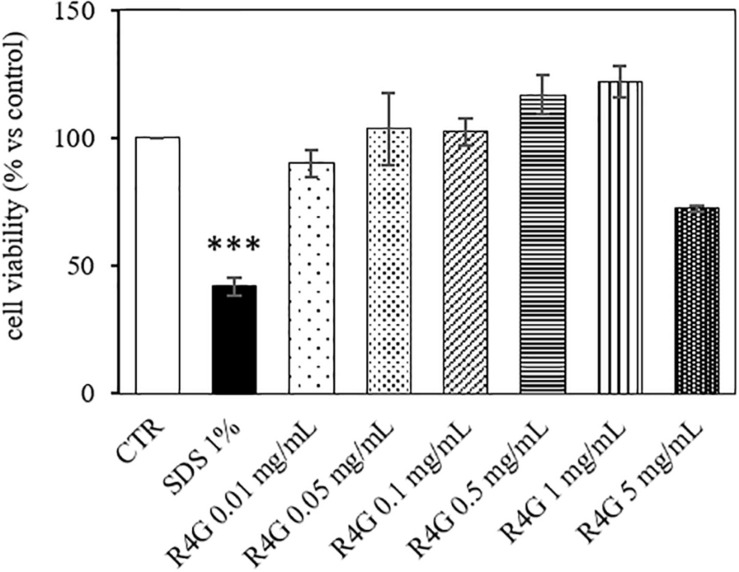
Viability of J774A.1 cells after exposure to R4G extract. Data are means ± standard errors [*n* ≥ 3, ***p < 0.001 vs. control (CTR) based on one-way ANOVA followed by Bonferroni *post hoc* test].

**FIGURE 2:**
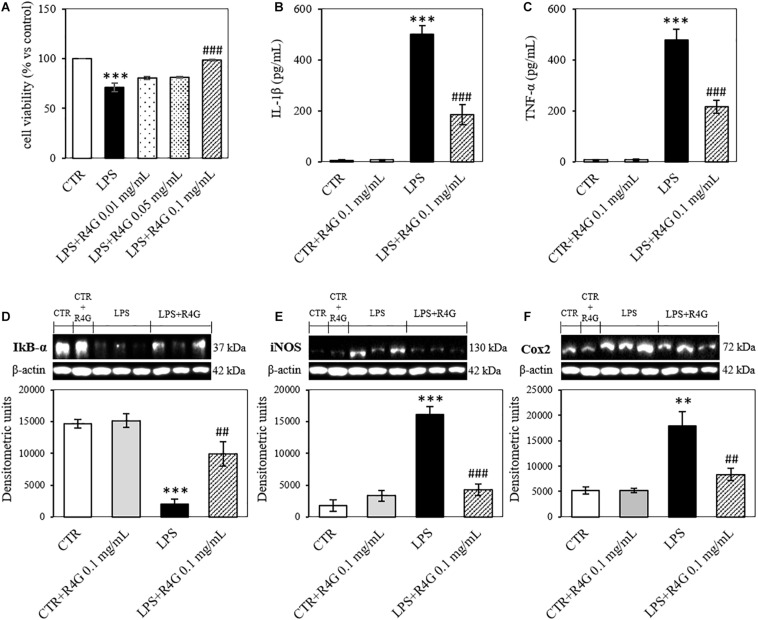
Anti-inflammatory activity of R4G in J774A.1 cells. **(A)** Cell viability after treatment with bacterial lipopolysaccharides (LPSs) with or without subsequent exposure to R4G extract. **(B)** Detection of the pro-inflammatory cytokine IL-1β in the culture supernatant. **(C)** Detection of the pro-inflammatory cytokine TNFα in the culture supernatant. Western blot analysis of cell lysates for the detection of **(D)** iNOS, **(E)** COX-2, and **(F)** IκBα. Data are means ± standard errors [*n* ≥ 3, ^##^*p* < 0.01; ^###^*p* < 0.001 vs. LPS; ^∗∗∗^*p* < 0.001, ^∗∗^*p* < 0.01 vs. control (CTR) based on one-way ANOVA followed by Bonferroni *post hoc* test]. Densitometric units = OD × mm^2^.

J774A.1 cells were exposed to R4G extract at various concentrations to determine the intrinsic toxicity of the extract and the concentrations suitable for subsequent testing (Figure 1). Once determined that the extract was not toxic even at higher concentration, we carried out all subsequent experiments with exposure concentrations of 0.01, 0.05, and 0.1 mg/mL, since, from a biotechnological point of view, the activities observed at low concentration are more interesting and more suitable for industrial exploiting. To determine the potential anti-inflammatory activity of the R4G extract, we treated the J774A.1 cells with 1 μg/mL LPS to induce an inflammatory response before adding the R4G extract. This revealed a significant protective effect conferred only by the highest concentration of extract we tested (0.1 mg/mL) (Figure 2A). Thus, we decided to use this concentration in the following experiments. To investigate the underlying mechanism, we measured the levels of the pro-inflammatory cytokines TNFα and IL-1β in the cell supernatant by ELISA. This revealed an increase in the production of both cytokines in response to LPS but a significant reduction when the cells were subsequently treated with R4G extract (Figures 2B,C). We also measured the expression of IκBα, iNOS, and COX-2 by western blot. This showed that LPS significantly induced the degradation of IκBα but the presence of R4G extract at a concentration of 0.1 mg/mL prevented the LPS-induced degradation of this molecule (Figure 2D). The pro-inflammatory enzymes iNOS and COX-2 were also induced by stimulation with LPS, but the levels of both enzymes were reduced significantly in the presence of R4G extract (Figures 2E,F).

#### Antioxidant Activity of RG4 Extract

**FIGURE 3:**
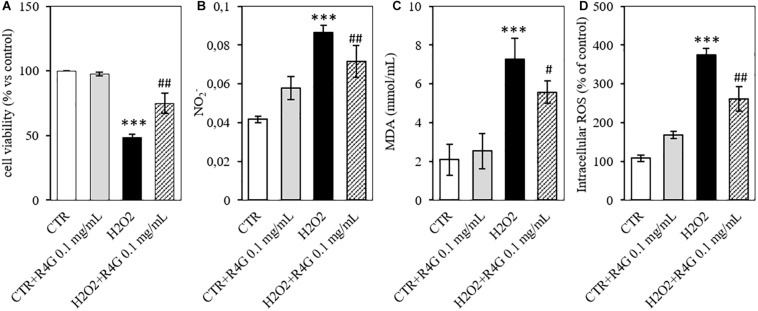
Antioxidant activity of R4G in J774A.1 cells. **(A)** Cell viability after H_2_O_2_ damage with or without pre-treatment using R4G extract. **(B)** Nitrite levels in the culture supernatant. **(C)** Intracellular level of malondialdehyde (MDA). **(D)** Intracellular level of reactive oxygen species (ROS). Data are means ± standard errors [*n* ≥ 3, ^##^*p* < 0.01; ^#^*p* < 0.05 vs. H_2_O_2_; ***p < 0.001, **p < 0.01 vs. control (CTR) based on one-way ANOVA followed by Bonferroni *post hoc* test].

To determine the potential antioxidant activity of the R4G extract, we pre-treated J774A.1 cells with 0.1 mg/mL of the extract before adding 200 μM H_2_O_2_ to induce oxidative stress. We found that the R4G extract again conferred a significant protective effect, increasing the viability of the cells in the MTT assay (Figure 3A). Moreover, the amount of nitrite released into the culture medium after H_2_O_2_ treatment was also significantly reduced in the pre-treated cells (Figure 3B). We showed that the R4G extract also reduced the accumulation of MDA, one of the products of membrane lipid peroxidation triggered by H_2_O_2_, indicating that the extract protects cellular lipids from induced oxidative damage (Figure 3C). Finally, we found that the pre-treatment also reduced the quantity of intracellular ROS present after H_2_O_2_ treatment (Figure 3D).

### R4G Extract Limit NF-κB Translocation and Induce the Expression of VEGF-A in Tissue Models

**FIGURE 4:**
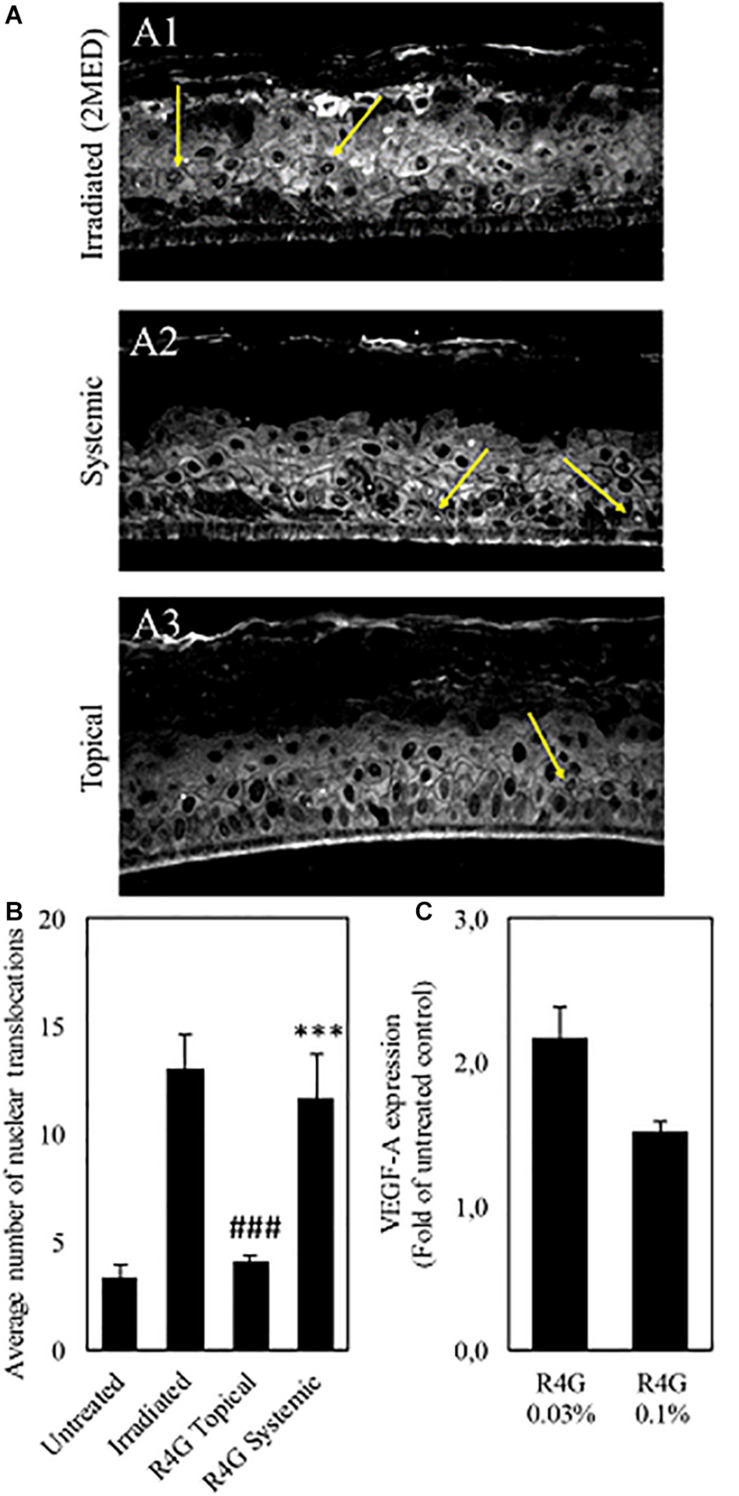
**(A)** Immunostaining against NF-κB (yellow arrows) on irradiated RHE (A1), on RHE after systemic (A2) and topical (A3) treatment with R4G extract. **(B)** Quantification of NF-κB nuclear translocation signal. The signal is significantly higher on irradiated control compared to untreated RHE. Topical exposure with R4G extract has significantly reduced NF-kB translocation compared to irradiated while systemic treatment has no significant effect vs. the irradiated control. **(C)** Modulation of VEGF-A gene expression after 72 h treatment relative to untreated control. Data are means ± standard errors (*n* ≥ 3; ###*p* < 0.001 vs. irradiation, ***p < 0.001 vs. untreated, based on Student’s *t*-test). MED, minimal erythema dose.

The anti-inflammatory mechanisms of the RG4 extract were investigated further by measuring the nuclear translocation of NF-κB in a human epidermal tissue model (RHE) and the expression of VEGF-A in a human microdermal model (HMEC). In the RHE model, UV irradiation was used to induce oxidative stress, which triggers the release of NF-κB from IκB and promotes nuclear translocation. The RG4 extract was applied at a concentration of 1 mg/mL to mimic either topical or systemic delivery. We found that nuclear translocation following irradiation was unaffected by the systemic application of RG4 extract but was significantly inhibited by topical application (Figures 4A,B). In the HMEC model, VEGF-A is involved in the formation and modulation of the dermal capillary network and is secreted by fibroblasts to improve the microcirculation. The RG4 extract resulted in a significant upregulation of *VEGF-A* gene expression, with the most potent effect observed at a concentration of 0.3 mg/mL (Figure 4C). A summary of the observed R4G extract activities is shown in Table 2.

**TABLE 2:** Activities of R4G extract.

**R4G effect**	**Tester system**	**Activity**
Prevention of LPS-induced inflammation	Mouse J774A.1 cells	- Cell viability - Quantification of TNFα and IL-1β Protein - Expression of IκBα, iNOS, and COX-2
Prevention of UV-induced oxidation	Human epidermal tissue model (RHE)	Inhibition of NF-κB nuclear translocation
Pro-angiogenic effect	Human microdermal model (HMEC)	Relative mRNA quantification of VEGF-A
Prevention of H_2_O_2_-induced oxidation	Mouse J774A.1 cells	- Cell viability - Nitrite-release measure - Lipid-peroxidation measure - Intracellular ROS measure

### Targeted and Untargeted Metabolomics Analysis Reveals Comparable Composition Between R4G Extract and Commercial Natural Red Carrot Extracts

The R4G extract showed strong anti-inflammatory and antioxidant activities in animal cell culture and could be suitable for the production of active ingredients for cosmetic or nutraceutical purposes. We therefore compared the metabolome of the R4G cells with that of three commercial products (CP1, CP2, and CP3; two powders and one juice) prepared, according to the manufacturers, from natural red carrot extracts.

**FIGURE 5:**
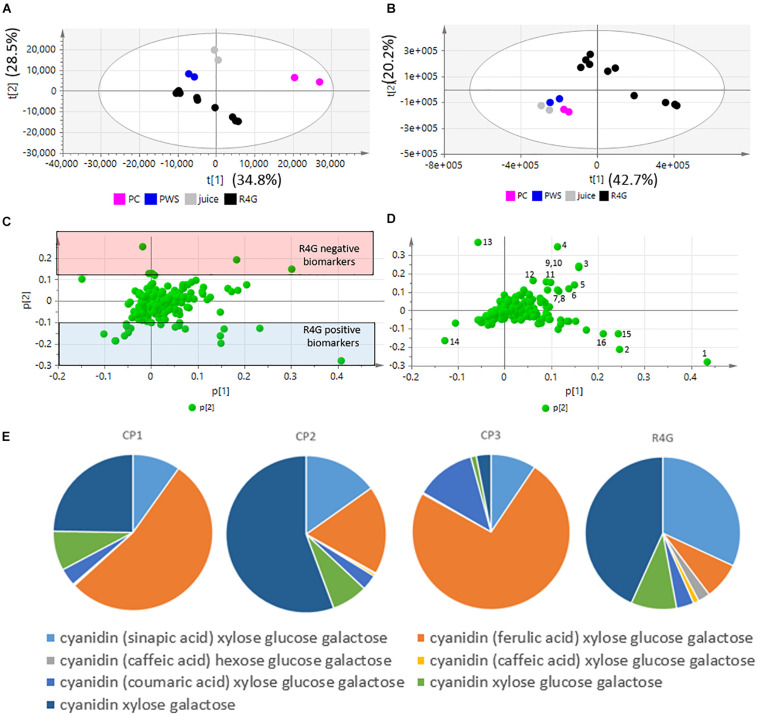
Untargeted metabolomics dataset, principal component analysis (PCA) and relative quantification. **(A,B)** PCA score plots. **(C,D)** PCA loading plots. **(A,C)** Raw data (sample diluted according to anthocyanin content). **(B,D)** Normalized data. Black dot = R4G extract; fucsia dot = commercial product (CP)1; blue dot = CP2; gray dot = CP3 (red carrot juice). **(E)** Anthocyanin profile, normalized data.

Five independent batches of R4G cells and the commercial products were analyzed by LC-MS. Given the different nature of the samples (fresh carrot cells, two dry carrot powders, one carrot juice), we diluted them as necessary to achieve approximately the same anthocyanin concentration in all extracts (Table 1). The LC-MS data were processed to generate the *m/z* feature data matrix (Supplementary File 1) and principal component analysis (PCA) was applied to find potential biomarkers of each extract, and to identify differences between the extracts derived from cultured cells and whole plants (Figure 5). The PCA score plot showed that the CP1 extracts differed from all the others (including R4G) along principal component 1 (PC1), whereas CP3 (juice) was separated from some of the batches of R4G extract along PC2 (Figure 5A). In order to find biomarkers responsible for the separation of some R4G extract from the juice, we chose 0.1 as reasonable threshold for P2 loading, thus considering *m/z* features with P2 < −0.1 as R4G positive biomarkers and *m/z* features with P2 > 0.1 as R4G negative biomarkers (Figure 5C and Supplementary File 1). The R4G positive biomarkers included sucrose, some anthocyanins, malic acid, and one hydroxybenzoic acid, whereas the negative biomarkers included citric acid, dicaffeoyl daucic acid, and caffeoyl quinic acid (Figure 5C and Supplementary File 1a). When the LC-MS relative quantitation data were normalized for the dilution factor, and (for the cells) also for the difference between fresh and dry weight (100 g fresh weight = 5.7 g dry weight in this cell line), the R4G cell extract were still mainly characterized by larger amounts of certain anthocyanins, malic acid, sucrose and one hydroxybenzoic acid (Figure 5D and Supplementary File 1b). However, the differences were merely quantitative (higher levels in R4G), as shown in Supplementary Figure 1. Furthermore, the anthocyanin profile showing the proportional distribution of the seven main carrot anthocyanins was also highly variable among the commercial samples (Figure 5E), with cyanidin (ferulic acid) xylose-glucose-galactose clearly dominant in CP3 and, to a lesser extent, in CP1, and cyanidin xylose-galactose dominant in CP2, and also R4G (Figure 5E). CP2 was the commercial extract with the greatest similarity to R4G, but the latter was characterized by a slightly higher ratio of acylated to non-acylated anthocyanins, a higher proportion of anthocyanin acylated with sinapic acid, and a lower proportion of anthocyanin acylated with ferulic acid (Figure 5E).

**FIGURE 6:**
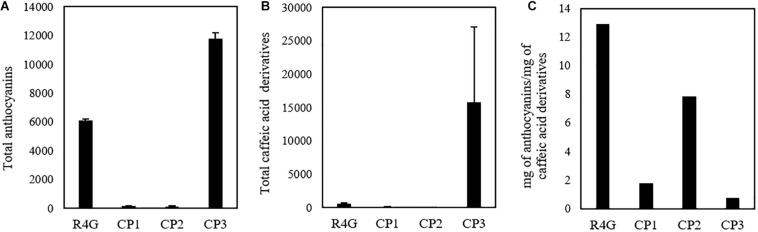
Quantification of total anthocyanins **(A)** and caffeic acid derivatives **(B)** and their ratio **(C)** in R4G extract, commercial product (CP)1, CP2, and CP3. Data are means ± standard deviations (*n* = 3), expressed as mg/100 g of dry weight (or mg/100 mL for CP3).

Given that untargeted MS analysis can only show the relative quantification of analytes, we determined the absolute quantities of the two major classes of metabolites (caffeic acid derivatives and anthocyanins) by HPLC-DAD analysis (Figure 6). The total quantity of caffeic acid derivatives in the R4G extract was similar to that of CP3 (but not CP1 or CP2), whereas the total quantity of anthocyanins was similar to that of CP1 and CP2 (Figures 6A,B). The R4G cell line has been selected for its ability of accumulate anthocyanins, thus explaining its higher ratio of anthocyanins to caffeic acid derivatives (Figure 6C).

### Anthocyanins in the R4G Extract Show Enhanced Stability

**FIGURE 7:**
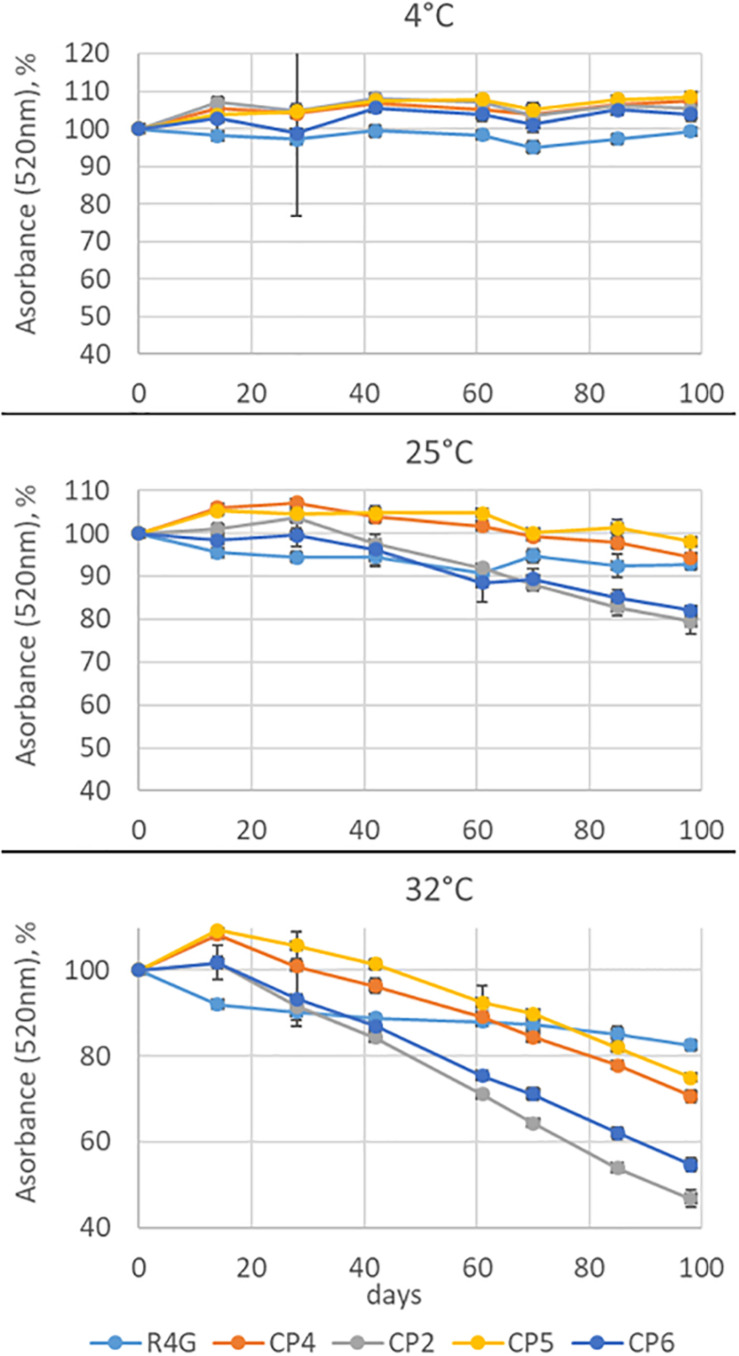
Anthocyanin stability time-course experiment at three temperatures. Absorbance was measured at 520 nm for 98 days. The absorbance is expressed as average percentage compared with the measurement on day 0. Data are means ± standard deviations (*n* = 3).

Finally, we tested the stability of the coloring conferred by the R4G extract by measuring its absorbance at 520 nm (the maximum absorbance wavelength of carrot anthocyanins) over 98 days compared with three commercial red carrot extracts (CP2, CP4, and CP5) as well as the commercial elderberry extract CP6. The extracts were stored at three temperatures (0, 25, and 32°C) for 3 months (Figure 7). There was no significant change in absorbance at 4°C for any of the extracts. At 25°C, we observed a 20% decrease in absorbance for extracts CP2 (carrot) and CP6 (elderberry) but no significant change in the others. At 32°C, we observed a continuous decrease in absorbance for all the commercial extracts, with CP2 and CP6 again showing the greatest sensitivity and losing ∼50% of their absorbance by the end of the experiment, whereas CP4 and CP5 lost only ∼30%. In contrast, the R4G extract showed a more rapid loss of stability than the commercial extracts for the first 20 days at 32°C, but then showed little further change and the overall decrease in absorbance after 98 days was < 20% making it the most stable overall (Figure 7).

## Discussion

The anthocyanin-producing cell line R4G was generated from the non-pigmented cell line K1 (initially derived from the commercial carrot variety Flakkese) by isolating rare pigmented cell clusters on the callus surface and forming pigmented cell lines (Ceoldo et al., 2005), and then selecting the R4G cell line from one of these pigmented cell lines (R3G) due its unusual ability to accumulate anthocyanins in the dark (Ceoldo et al., 2009). The R4G cell metabolome is mainly characterized by the presence of anthocyanins, hydroxycinnamic acid derivatives, and hydroxybenzoic acid derivatives (Ceoldo et al., 2009; Toffali et al., 2013). The anthocyanins are mainly based on cyanidin and accumulate as both acylated and non-acylated versions, whereas the hydroxycinnamic acids are mainly derived from caffeic acid. Anthocyanins are well-known plant pigments used as food colorings, pharmaceutical ingredients, and health-promoting additives (Khoo et al., 2017). They accumulate *in vivo* as glycosides, often acylated by aliphatic or aromatic organic acids, particularly phenolic acids (Zhao et al., 2017). Glycosylation and acylation influence their solubility and stability (Khoo et al., 2017; Zhao et al., 2017). The stability of acylated anthocyanins depends on the types, numbers and positions of acyl groups, and aromatic acyl groups confer much greater stability than aliphatic ones by forming intramolecular co-pigment complexes (Zhao et al., 2017). In our experiments, the R4G anthocyanins were much more stable over time than enocyanins (from red-skinned grapes) and elderberry anthocyanins, reflecting the higher content of acylated anthocyanins in the R4G extract but the prevalence of non-acylated anthocyanins in elderberries and grapes (Anesi et al., 2015; Da Silva et al., 2019). Surprisingly, the anthocyanins in the R4G extract were also more stable than natural red carrot anthocyanins. This requires further investigation, but it is possible that the phenomenon may reflect the combined properties of the entire phytocomplex. Given that we selected the R4G cell line for its ability to accumulate anthocyanins, the relevant difference between R4G and natural red carrot extracts is the higher ratio of anthocyanins to caffeic acid derivatives in the R4G extract. This could also explain the difference in potency between the R4G extract and commercial counterparts because anthocyanins are much stronger antioxidants than hydroxycinnamic acids (Rice-Evans et al., 1997).

The bioavailability of anthocyanins is low when administered as pure compounds, but increases greatly when they are consumed as natural phytocomplexes, as in blackcurrant and mixed red fruits (Miyazawa et al., 1999; Matsumoto et al., 2001; Czank et al., 2013). Anthocyanins are proposed to offer many potential health benefits due to their antioxidant activity, including the promotion of angiogenesis and cardiovascular health, neuroprotection, the inhibition of cancer, diabetes, and obesity, the improvement of visual health, and activity against pathogens (Khoo et al., 2017). Similarly, caffeic acid and its derivatives are antioxidants that confer the same health benefits as anthocyanins, as well as anti-inflammatory/immunostimulatory properties, the prevention of atherosclerosis, and protection of the liver (Magnani et al., 2014; Espíndola et al., 2019). The bioavailability and pharmacokinetics of such compounds have been studied in detail (Espíndola et al., 2019).

### Multifunctional Phytocomplexes From R4G Cells Are Promising as Cosmetic and Nutraceutical Ingredients

Freeze-dried R4G extract were tested for potential activity in the mouse macrophage/monocyte cell line J774A.1 and in two *in vitro* human skin models (RHE and HMEC). The topical application of R4G extract to RHE provides a good model for cosmetic applications because RHE forms a stratified epithelium with similar biochemical and physiological properties to human skin *in vivo*, thus representing the current most promising alternative to animal experiments, *ex vivo* explants and submerged cell monolayers (Gordon et al., 2015; Zhuang, 2016). The biological relevance and prediction power of the models used reflect the presence of an organized tissue with different living cell layers allowing us to assess topical products at realistic clinical doses and under typical exposure conditions. *In vitro* reconstructed human skin models are relevant test systems to assess product efficacy because they replicate responses in terms of gene expression, signal transduction, and crosstalk via the production of soluble mediators and key biomarkers. Specifically, we used a stress model based on the UV irradiation of RHE to test for antioxidant and anti-inflammatory properties and a dermal microtissue assay to understand the effects of our extract on primary dermal fibroblasts in a 3D tissue context. These models revealed that the R4G extract inhibited the nuclear translocation of NF-κB and triggered a pro-angiogenic effect by inducing the expression of VEGF-A, both of which are desirable responses for cosmetic and nutraceutical ingredients.

NF-κB is a cytoplasmic sensor that is usually inhibited by binding to IκB proteins. Various internal and external danger signals associated with senescence and aging cause the disassociation of this complex, exposing the nuclear localization signal of NF-κB and facilitating translocation (Adler et al., 2007). In the nucleus, NF-κB acts as a transcription factor to activate genes encoding multiple pro-inflammatory cytokines, chemokines, adhesion molecules, growth factors, metallo-proteinases, and enzymes that produce signaling molecules such as eicosanoids and nitric oxide (Gilmore and Wolenski, 2012). An inducible genetic blockade of NF-κB for 2 weeks in the epidermis of mice that were chronologically aged by expressing a dominantly active version of IκB reverted the tissue characteristics and global gene expression profiles to those of young mice (Adler et al., 2007). Our results suggest that the topical application of R4G extract may slow the aging process by inhibiting the nuclear translocation of NF-κB.

There are five members of the VEGF family in mammals: VEGF-A, VEGF-B, VEGF-C, VEGF-D, and placental growth factor (PlGF). VEGF-A is the best characterized member (Peach et al., 2018) and is the principal regulator of angiogenesis under normal physiological conditions and in the context of disease (Carmeliet, 2005). Treatment with exogenous angiogenic factors has been shown to restore normal capillary density in experimentally-depleted areas and the same approach has been proposed as a strategy to improve function in areas of naturally impaired microcirculation (Ambrose, 2017). Capillary density has been shown to decline with age in the skin (Helmbold et al., 2006), larynx (Russell et al., 2008), colon (Gabella, 2001), and kidneys (Stefanska et al., 2015) and correlates with a general depletion of angiogenic factors. Rupnick et al. (2002) suggested that the age-linked decline of angiogenesis in the skin may promote wrinkling due to the depletion of subcutaneous fat. We found that the systemic administration of R4G extract induced the expression of VEGF-A in our co-culture microtissue model, thus we hypothesized that the described extract has the potential to promote angiogenesis and reactivate the microcirculation by increasing the capillary density.

For nutraceutical applications of the described extract, there is growing evidence that anthocyanins have numerous beneficial effects in the context of cardiovascular health, protection against cancer, and the prevention of diabetes and obesity in preclinical studies of mice reared on high-fat diets (Cerletti et al., 2017). Several mechanistic hypotheses have been proposed, including the direct scavenging of free radicals to reduce oxidative stress, the prevention of lipid peroxidation, and thus the suppression of signaling pathway leading to cell proliferation and apoptosis (Khoo et al., 2017). In the context of cardiovascular disease, the antioxidant activity of anthocyanins has been shown to block nitric oxide production and promote the activity of the transcription factor Nrf2, which in turn regulates the expression of multiple genes encoding proteins that protect against oxidative damage, such as heme oxygenase-1 (Reis et al., 2016). The systemic anti-inflammatory activity of anthocyanins may also reflect their ability to modulate the gut microbiome, increasing the prevalence of beneficial organisms such as bifidobacteria (Morais et al., 2016). However interventional studies in humans are more complex than *in vitro* models, so the observed beneficial effects cannot be translated immediately into the improvement of risk biomarkers for chronic disease unless healthy subjects are deliberately placed under stress, such as the provision of fatty meals (Cerletti et al., 2017).

### The R4G Extract Could Be Safe for Human Consumption and Its Secondary Metabolome Shows Greater Stability Than That of Commercial Carrot Extracts

Plant cell and tissue culture technology allows plant biomass to be grown under sterile conditions and in artificial completely-defined media, with none of the environmental harm associated with conventional agriculture. The living biomass is uncontaminated with pesticides or heavy metals, and has never been in contact with animal-derived components (Eibl et al., 2018). Importantly, cells cultured *in vitro* retain many of the metabolic properties of the species they represent, and are therefore suitable for the production of fine chemicals, pharmaceuticals, nutraceuticals, cosmetic ingredients, and even complete foods, as in the emerging concept of “cell agriculture” either in central facilities or in the home (Barbulova et al., 2014; Eibl et al., 2018; Nordlund et al., 2018).

Plant cell biomass used as food or for the preparation of nutraceutical supplements must be safe for human consumption. The metabolic potential of plant cells, tissues and organs is under genetic control, and when a cell culture is derived from a species that is traditionally consumed as food, it is natural to assume the cells will also be safe. However, all food crops have edible and non-edible parts, and some edible parts (such as fruits and seeds) may only become edible at a certain stage of development. The accumulation of secondary metabolites likewise depends on the developmental stage and tissue. Plant cells are typically derived from undifferentiated callus tissue and they are cultivated in an artificial environment, so equivalence cannot be drawn to the whole plant. Thus, it is important to compare the cell culture extract composition with plant extract.

We used untargeted metabolomics based on LC-MS to survey the metabolome of the R4G extract and similar commercial extracts, combined with a quantitative targeted metabolomics approach, automatic data processing and a combination of univariate and multivariate statistics. This showed that the R4G extract is richer in anthocyanins than the other sources but the levels of other metabolites are similar in the R4G and commercial extracts. This is unsurprising given that we selected the R4G line on the basis of its high anthocyanin content. These results suggest that R4G extract may be safely consumed by humans as nutraceutical supplement.

Natural products used for the industrial production of cosmetic ingredients and nutraceutical supplementation must be stable to ensure a long shelf life. The R4G extract proved to be more stable than four commercial products (three derived from red carrot and one from elderberry). Color stability depends on many factors, including the structure and concentration of the pigments, pH, temperature, light intensity and quality, co-pigments, metal ions, and the presence and abundance of metabolites such as ascorbic acid (Bakowska-Barczak, 2005). However, our tests were carried out under standardized conditions of temperature, light exposure, and pigment concentration, so any differences in stability must depend solely on the composition of the extract. The color stability conferred by anthocyanins also depends on the specific chemical structure, because certain modifications increase stability (e.g., hydroxylation) whereas others have the opposite effect (e.g., methylation). Stability is also affected by the nature of any glycan groups (e.g., anthocyanins containing galactose are more stable than those containing arabinose). Finally, the acylation profile is also an important factor: acylated anthocyanins are more stable than non-acylated ones, aromatic acyl groups confer greater stability than aliphatic groups, and acylation on ring B confers greater stability than acylation on ring A (Bakowska-Barczak, 2005). Our data broadly agree with these observations. For example, the CP2 extract was closest in composition and behavior to R4G and can therefore be defined as the most “substantially equivalent.” Even so, R4G was more stable than CP2 and this may in part reflect the higher proportion of acylated anthocyanins in the R4G extract.

## Conclusion

We tested an extract produced from the pigmented carrot cell line R4G to determine its suitability as a cosmetic ingredient and nutraceutical food additive. We found evidence for antioxidant and anti-inflammatory activity in mouse J774A.1 cells exposed to bacterial LPS and H_2_O_2_, as well as an anti-aging effect (suppression of NF-κB translocation) and the ability to promote microcirculation (induction of VEGF-A expression) in two *in vitro* human skin models. The high content of anthocyanins in the extract suggested an additional application as a natural food colorant, and we found that the R4G extract is very similar to commercial extracts, but its higher content of acylated anthocyanins increases its color stability. HR-UPLC-MS and HPLC-DAD-MS analysis of the R4G metabolome followed by multivariate statistics allows us to define the content of the R4G extract and confirm its comparable composition with extracts from natural sources. We think that these plant biomass cultivated in bioreactor could provide a safer and more standardized approach for production of ingredients used in the cosmetic, nutraceutical and food sectors. Moreover, we are now investigating the suitability of these systems for production of high added value but low concentration plant chemicals through metabolic engineering and subsequent metabolite purification. Since the plant cell lines grow in the confined and protected bioreactor environment, they could represent environmentally safe and law compliant systems for metabolic engineering, thus greatly increasing the range of metabolites that can be produced with this technology.

## Data Availability Statement

All datasets generated for this study are included in the article/Supplementary Material.

## Author Contributions

MB performed the stability and targeted metabolomics experiments. LC performed the biological activity on RHE and on microdermis (co-culture of human primary dermal fibroblasts with human microvascular dermal endothelial cells). SC and ES performed the anti-inflammatory and antioxidant activities on J774-A1 monocyte/macrophage cell line using dry R4G extract. OB, CG, and GP performed the R4G cell line maintenance and growth and prepared the dry R4G extract from cell suspensions. MC and FG performed the untargeted metabolomics experiments. EB and AS contributed to the experimental design of the evaluation of biological activity on RHE and on microdermis (co-culture of human primary dermal fibroblasts with human microvascular dermal endothelial cells). SN, MC, ES, and GP contributed to writing some sections of the manuscript. GP and ES contributed with the critical reading of the manuscript. FG and GP coordinated the research. FG and LA wrote the manuscript. SN revised the statistics and organized the figures of the manuscript. All the authors approved the manuscript.

## Conflict of Interest

MB, GP, ES, OB, CG, and BE were employed by the company Demethra Biotech Srl. LC was employed by the company VitroScreen Srl. The remaining authors declare that the research was conducted in the absence of any commercial or financial relationships that could be construed as a potential conflict of interest.

## Footnotes

1 http://mzmine.github.io/download.html

## Supplementary Material

Supplementary Figure 1 Relative abundance of metabolites in the R4G carrot cell extract. Data are means ± standard deviations (*n* = 3).
