# Supplementary figures and images for: Red Carrot Cells Cultured in vitro Are Effective, Stable, and Safe Ingredients for Skin Care, Nutraceutical, and Food Applications
Source: Front Bioeng Biotechnol. 2020 Oct 21;8:575079. doi: 10.3389/fbioe.2020.575079 (PMC7609948; doi:10.3389/fbioe.2020.575079)

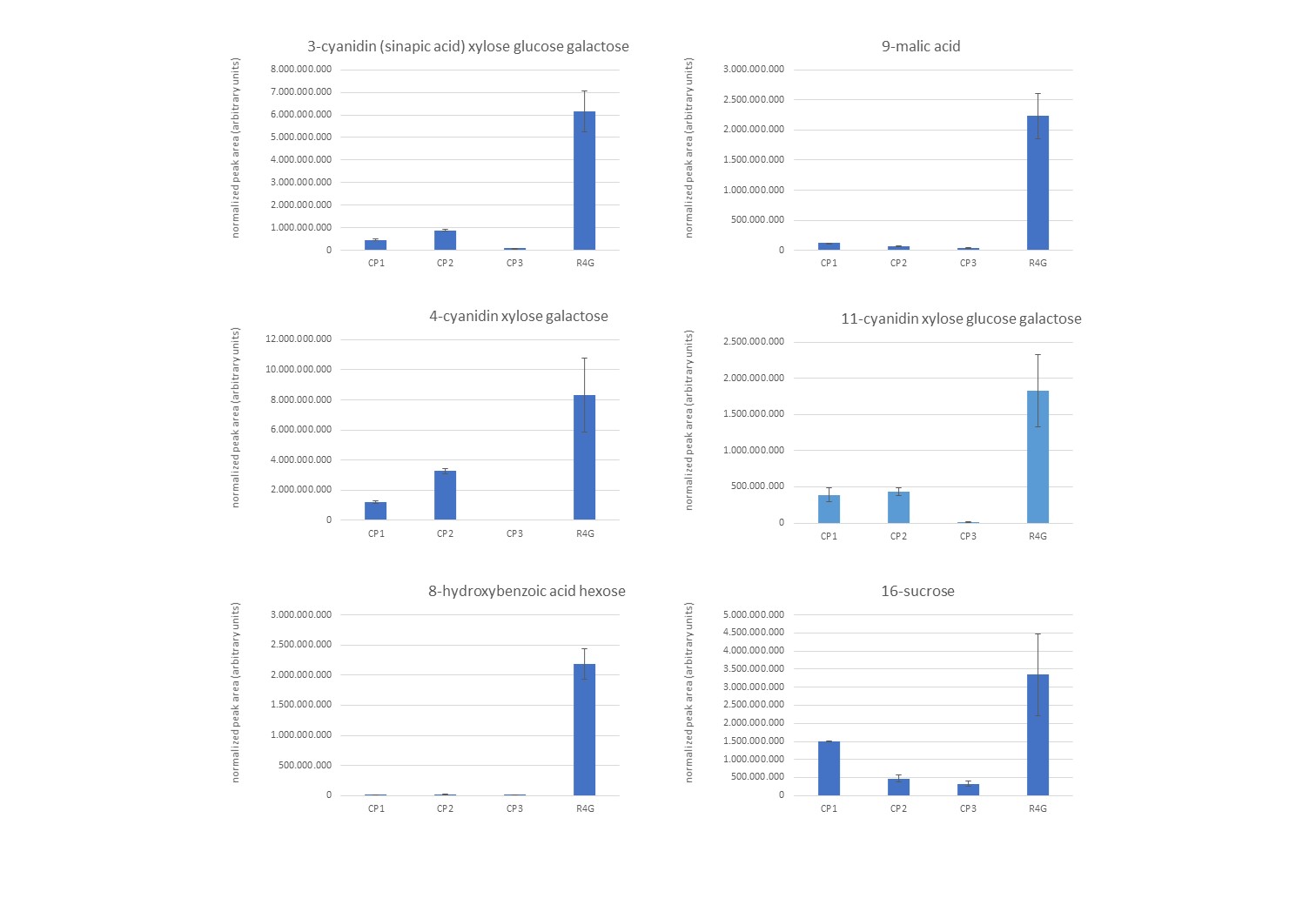

Supplement: Supplementary Figure 1 — Relative abundance of metabolites in the R4G carrot cell extract. Data are means ± standard deviations (n = 3). [file Image_1.jpg]
